# Editorial: Constructing objectivity: emotions in legal decision-making

**DOI:** 10.3389/fsoc.2025.1639607

**Published:** 2025-07-15

**Authors:** Louise Victoria Johansen, Mojca M. Plesničar, Sharyn Roach Anleu, Stina Bergman Blix

**Affiliations:** ^1^Faculty of Law, University of Copenhagen, Copenhagen, Denmark; ^2^Institute of Criminology at the Faculty of Law, University of Ljubljana, Ljubljana, Slovenia; ^3^College of Humanities, Arts and Social Sciences, Flinders University, Adelaide, SA, Australia; ^4^Department of Sociology, Uppsala University, Uppsala, Sweden

**Keywords:** objectivity, court, legal decision-making, empathy, emotion, rationality

## 1 Introduction

The field of Law and Emotion originated in discussions that disputed the bifurcation of emotion and law and inspired, among other topics, socio-legal research on the dynamics and social dimensions of court work. The field has grown exponentially in diverse disciplines such as sociology, law, anthropology, criminology, and philosophy. This Research Topic originated from a symposium outside of Stockholm, Sweden, during September 2023. The symposium was part of the dissemination of the European Research Council project JustEmotions. It gathered prominent and promising scholars with expertise in different legal systems with the overall aim of engaging in stimulating cross-cultural discussions on empirical research in and around courts. As a result of these discussions, we have put together a Research Topic of empirical studies within the field of law and emotion, with contributions from Argentina, Australia, Denmark, Italy, the Netherlands, Poland, Portugal, Slovenia, and Sweden.

At the core of legal decision-making lies the fundamental principles of objectivity, impartiality, and independence. The Research Topic, *Constructing Objectivity—Emotions in Legal Decision-making*, addresses the dialectical processes of translating these principles into everyday judicial practice across a wide range of legal systems.

The Western, modern understanding of objectivity characterizes it as opposite to subjectivity; objectivity incorporates knowledge that “bears no trace of the knower” (Daston and Galison, 2010, p. 17). Objectivity is usually associated with science, in particular the natural sciences and requires the (systematic) observation of things that become facts (Fuchs and Ward, 1994). It does not matter who observes, the facts are observable, directly or indirectly; discoveries are made, and truth can be found. Empirical reality exists independently of the observer. The term objectivity “can be applied to everything from empirical reliability to procedural correctness to emotional detachment” (Daston and Galison, 1992, p. 82).

Objectivity becomes a disembodied state of being, not dependent on (subjective) interpretation and previous experience. Several concepts related to objectivity, often used synonymously and interchangeably—dispassion, independence, impartiality, neutrality—all suggest an “unbiased, unprejudiced state of mind” (Geyh, 2013, p. 512 fn 96).

When legal professionals operationalize objective practice, they tend to link it to work processes free of bias and personal standpoints, incorporating standardization and typification (Rogers and Erez, 1999). In the actual work of legal professionals, objectivity is not a state of being but an ongoing process of balancing engagement and disengagement, commitment and detachment (Jacobsson, 2008; Roach Anleu and Mack, 2019; Bergman Blix and Wettergren, 2019). Building on empirical research, including observations, interviews, shadowing, vignettes and workshops with legal professionals, this Research Topic addresses objectivity in the making. It investigates such questions as: What role do court architecture and material objects play in the emotional dynamics of legal procedure? How do judges manage victims' emotional statements or evaluate their credibility? How do judges make independent decisions in a collective setting? What role does extra-legal (lay/specialist) expertise play in co-constructing legal knowledge relevant to decision-making? How do judges embrace the idea of objectivity? How does the growing digitalization of courts and hearings affect the answers to these questions? In so doing, the articles encompass the complexity of objectivity as an ideal, a judicial value, requiring performance, promoting courtroom atmosphere, and as sometimes feigned.

Ultimately, the question of whose emotions are acknowledged and to what effect, is not a neutral one. It is a mechanism of power, shaping how legal authority is performed, reinforced, and sometimes contested. By interrogating the emotional hierarchies embedded in legal practice, this Research Topic challenges the long-standing myth that objectivity is an absence of emotion. Instead, it reveals that objectivity is an emotionally managed ideal—one that legal professionals, victims, and even technological systems must continually work to perform and sustain.

This introduction is divided into four themes that together cover the different articles in the Research Topic. First, we discuss whose emotions we study and the implications for our analytical lens. Next, we turn to the role of space to co-create emotional dynamics, both looking at the architecture of courts and imaginary spaces for future virtual court hearings. Third, we focus on the role of emotion in rulings and sentencing, and lastly, we discuss how preconceptions and bias may create and uphold differences in legal settings.

## 2 Whose emotions do we study?

The study of emotions in legal decision-making requires fundamental inquiry into whose emotions are recognized, analyzed, and given epistemic value within judicial processes. Traditionally, the law has positioned itself as a domain of rationality, where emotions are viewed as external disruptions to objectivity (Grossi, 2019; Karstedt et al., 2011). However, socio-legal research increasingly demonstrates that emotions are not merely incidental but constitutive of legal practice—shaping interactions, influencing credibility assessments, and reinforcing institutional norms (Bandes et al., 2021; Bandes and Blumenthal, 2012; Maroney T. A., 2006; Nordquist and Blix, 2022). The question of whose emotions we study, then, is not just methodological but also deeply political, as it reflects broader power structures within the legal system (Rossmanith et al., 2024).

### 2.1 Expanding the analytical lens: from defendants and victims to legal professionals

A fundamental question in the study of law and emotion is whose emotions are assessed as relevant to legal decision-making. Traditionally, courtroom emotion has been studied in relation to victims and defendants, focusing on how their emotional expressions influence credibility assessments and legal outcomes (Maroney T., 2006; Tsoudis and Smith-Lovin, 1998; van Doorn and Koster, 2019). Although not a primary focus in the articles collected here, defendants' emotions have long been a central concern in both sociological and legal research, for instance examining how expressions of remorse, defiance, or emotional detachment influence assessments of credibility and sentencing outcomes (e.g., Field and Tata, 2023). Defendants' emotional perspectives remain crucial to understanding courtroom dynamics: what is perceived as objective or neutral from within the legal system may appear distant or alienating from the defendant's standpoint (Johansen, 2022). These divergences highlight how legal objectivity is not a universally shared experience but one that is often shaped by power and positionality. However, a broader approach reveals that legal professionals—judges, prosecutors, and even lay decision-makers—are also deeply embedded in emotional dynamics (Bergman Blix and Wettergren, 2018). Their emotions do not merely exist alongside judicial reasoning; they actively shape how legal objectivity is constructed, negotiated, and performed (Grossi, 2015).

One key issue is that the emotions of certain courtroom participants are deemed appropriate within legal settings, while others are treated as disruptive. As papers in this Research Topic show, victims, for example, are often expected to display their distress in ways that align with culturally embedded norms of believability (Johansen). The legal system does not simply react to these emotional expressions; it actively structures the terms on which emotions can be expressed. Similarly, defendants' emotions—whether remorse, defiance, or detachment—are frequently interpreted as indicators of moral character or legal responsibility. Judges play a crucial role in interpreting, managing and curating these displays, ensuring that emotions do not appear to unduly influence legal outcomes (Bosma).

At the same time, the emotions of legal professionals—particularly judges—are often framed as either non-existent or irrelevant to decision-making. The ideal of judicial neutrality suggests that legal actors should remain detached, prioritizing reason over feeling. Yet, in reality, judges and prosecutors engage in significant emotional labor, managing their own affective responses while navigating the emotions of others (Plesničar). Judicial deliberations, for example, are shaped by collective emotional dynamics such as trust, doubt, and confidence, all of which influence decision-making processes (Bergman Blix and Törnqvist).

Even lay decision-makers, such as jurors and lay judges, must reconcile their emotional responses with legal reasoning. The assumption that professional judges embody rationality while lay participants bring emotion and subjectivity reinforces a false dichotomy between expertise and emotion. In practice, lay participants often actively regulate their emotions, working to align their judgments with legal norms. This negotiation is shaped by their efforts to reconcile personal moral intuitions with the affective expectations embedded in courtroom procedure, revealing how legal objectivity is co-constructed through emotional reflexivity across professional and lay domains (Amietta).

By expanding the analytical lens to include the emotions of legal professionals and the institutional structures that regulate emotion, we move beyond simple models that treat emotion as a contaminant of legal reason. Instead, we see that objectivity itself is constructed through affective work—a process that is neither static nor individual but collectively negotiated.

### 2.2 The politics of emotional visibility: whose emotions matter?

If all courtroom actors engage in emotional labor, the next question is: whose and which emotions are acknowledged or rendered invisible? Courts do not merely respond to emotion; they produce hierarchies of emotional legitimacy, determining which feelings are recognized as relevant and which are dismissed, suppressed, or framed as bias.

Some emotions—such as judicial composure, prosecutorial confidence, and defense skepticism—are viewed as neutral and professional. Others, such as victim distress, judicial empathy, and lay skepticism of legal reasoning, are treated as potentially disruptive to legal objectivity (Johansen; Stepień). These distinctions are rooted in professional norms and reflect broader cultural and gendered expectations about how authority should be expressed and felt in legal settings. Emotional legitimacy is unequally distributed; expressions of empathy in judges or moral doubt in jurors, for instance, are often tolerated when tightly managed or reframed as cognitive stance-taking. In courtroom settings where evidentiary clarity is limited—such as rape trials or jury deliberations—these tensions become especially visible, revealing how legal actors must continuously calibrate their emotional positioning to maintain institutional legitimacy (Amietta; Bladini).

These hierarchies are particularly evident in how victims' emotions are managed within courtroom narratives. While legal procedures often claim to accommodate emotional testimony, this accommodation comes with strict limitations. Victims are expected to express emotions in institutionally appropriate ways—sufficiently distressed to appear credible but not overly emotional to the point of seeming irrational (Bosma).

Judges themselves are subject to conflicting emotional expectations. While legal norms dictate that they must remain unemotional and detached, they experience doubt, trust, frustration, and even empathy—emotions that influence their decisions in subtle but powerful ways (Bergman Blix and Törnqvist; Stepień). Judges develop strategies to manage these internal tensions, sometimes formalizing their approaches into unofficial rules and internalized frameworks that allow them to maintain a sense of emotional coherence in their rulings (Plesničar). These affective processes are especially visible in courtroom contexts where evidentiary ambiguity requires legal actors to draw on embodied and empathic forms of understanding. In such cases, objectivity is not merely about distancing oneself from emotion but about calibrating one's emotional responses in relation to others—an approach that foregrounds empathy as an epistemic practice, particularly in settings marked by gendered power dynamics (Bladini).

The control of empathy is a particularly contentious issue. While some judges see empathy as a cognitive tool that aids decision-making, others view it as a threat to impartiality. Legal professionals often frame their own emotional engagement in strategic ways, ensuring that it does not appear to compromise their authority (Stepień).

The politics of emotional visibility also extends beyond human actors. The rise of artificial intelligence (AI) in judicial decision-making introduces new ways of structuring emotional legitimacy. While AI is often framed as a neutral tool to reduce bias, it ultimately reflects and reinforces existing emotional hierarchies. Because these systems rely on historical data and legal precedents, they risk encoding the same biases that have traditionally shaped which emotions are recognized as relevant within the legal process (Contini et al.).

## 3 Court spaces and emotional dynamics

Courtrooms are prominent physical environments rich in symbolism, authority, and emotional resonance. These spaces not only shape users' experiences but are deeply entwined with legal decision-making. Interactions, whether between professionals and laypeople or among legal professionals, are influenced by the courtroom's architecture and the emotional, linguistic, and behavioral norms it imposes (Dahlberg, 2009).

It is no coincidence that defense and support attorneys coach their clients on appropriate courtroom behavior (Flower, 2019) or that legal professionals interpret subtle cues, such as facial expressions or a judge's weary pencil drop, as indicators of emotional states (Bergman Blix and Wettergren, 2018). This Research Topic brings together contributions that examine the courtroom as a restrictive emotional environment and site where emotions serve as tools for acquiring knowledge (Branco; Tait and Rossner).

Emotions provide judges and legal actors with insights about cases and people; a defendant's or victim's emotional outburst—or lack thereof—can impact judicial outcomes. What counts as an “appropriate” emotional display is shaped by both cultural and spatial expectations (Johansen**)**.

While courtrooms have attracted substantial attention in research on law and emotion, other areas within courthouses remain underexplored despite their critical role in decision-making. One such space is the deliberation room, for instance, in civil law jurisdictions where judges and lay judges engage in collective decision-making. This backstage setting becomes a site for negotiating between legal reasoning and everyday knowledge (Amietta), as well as for sharing and processing doubt. In the Swedish context, Bergman Blix and Törnqvist show how feelings of certainty and uncertainty are integral to the evaluation of case knowledge during deliberations characterized by a collective environment quite different from the formal courtroom setting. The study links shared attention, emotional energy, and trust to the success or failure of legal deliberations, highlighting how epistemic emotions shape legal decisions.

Expanding the scope to the courthouse as a symbolic and architectural co-creator of emotions, Branco demonstrates how the inadequate design and maintenance of Portuguese Family Courts affect emotional experiences for all users—judges, prosecutors, and litigants—alike. These emotionally charged spaces shape perceptions of fairness and empathy, with different users experiencing them according to their roles. Substandard courthouse conditions foster frustration and insecurity, undermining professionals' legitimacy and the quality of their decisions, while litigants may feel alienated or excluded from adequately participating in their own cases.

The definition of courtroom space is evolving through digitalization and the integration of Generative AI. Participants in legal proceedings may now join remotely, raising questions about how emotions are expressed and perceived in hybrid or virtual settings, redefining experiences of justice and transforming perceptions of trials (Flower, 2025; McKay, 2018; Rossner et al., 2021). Drawing on literature, dramaturgy, and the sociology of emotions, Tait and Rossner propose a framework for designing immersive judicial environments, showing how such spaces could enhance accessibility, empathy, inclusiveness, and procedural fairness, while raising questions about security and privacy. Rather than a static reproduction, the metaverse courtroom is presented as a flexible, performative space that challenges conventional notions of the physical courtroom.

More radically, aspects of sentencing can be delegated to generative AI. In an Italian criminal case context, Contini et al. highlight how predictive systems disrupt the emotive-cognitive foundation of legal judgment. AI tools simplify judicial processes by relying on statistical patterns, side lining the emotional nuances and interpretive reasoning that are crucial to fair outcomes. These systems diminish the interpersonal exchange such as those in deliberation rooms that are vital for constructing legitimate decisions, as emphasized by Bergman Blix and Törnqvist.

Since generative AI is hailed as a time-efficient and potentially more impartial tool, its absence of emotional engagement must be scrutinized in contrast to the dynamic, human-driven processes it aims to replace, even if those are not without their flaws.

## 4 Emotions, knowledge, and decision-making

Emotion is not simply present in legal decision-making—it is learned, rehearsed, and professionally managed. Judges and legal professionals are socialized into emotional repertoires that align with institutional expectations of neutrality, detachment, and control. Emotional expressions must be calibrated to fit the normative frameworks of the legal field, often through tacit training, observation, and collective practice as well as through formal training and professional education (Bergman Blix and Wettergren, 2018; Roach Anleu and Mack, 2014). Emotional management is not a deviation from legal reasoning, rather an essential part of it, constituting how legal actors embody authority, build trust, and navigate uncertainty. This section examines how emotional competencies are cultivated and performed within the judiciary and legal professions.

Legal professionals are acutely aware of the importance of consistency in sentencing. Yet, disparities often arise due to subjective influences such as emotions, biases, and differing cognitive approaches. In Slovenia, Plesničar illustrates how legal professionals attempt to reconcile the ideal of objectivity with the inherent imperfections of human judgment. Strategies include informal peer discussions, *ad hoc* sentencing guidelines, and other support mechanisms. These efforts often generate emotional strain and are not always successful in creating consistency. Nonetheless, legal professionals resist systemic reforms like mandatory sentencing due to concerns about preserving judicial discretion, independence and individualized justice, calling for a nuanced approach to sentencing that considers both emotional and systemic dimensions.

Judicial work inherently involves managing complex emotional dynamics, making emotional competence a critical, though often unspoken, part of performing judicial authority (Roach Anleu et al., 2021). A recurring theme across several contributions is the ambivalence legal actors feel in balancing emotional engagement with judicial objectivity. For instance, judges in Poland actively manage this tension by distributing empathy evenly among parties and regulating their emotional expressions in the courtroom (Stepień). Empathy, in this context, is simultaneously viewed as a valuable resource and a potential threat to impartiality. Its legitimacy depends on how it is framed and applied. In the Netherlands, Bosma examines the expanded use of Victim Impact Statements in criminal proceedings since 2016. While these statements raised concerns about judicial bias, judges have sought to maintain objectivity by acknowledging victims' emotions empathetically, while fostering empathy between victims and defendants.

Whereas legal professionals strive to moderate emotionality in service of objectivity, lay judges often face the inverse stereotype as overly emotional and thus must be restrained (Johansen, 2019). In Argentina, lay participants in mixed tribunals challenge this characterization. As Amietta shows, these lay actors see themselves not merely as emotional or common-sensical contributors but also as engaged co-users of legal language and reasoning. From both professional and lay perspectives, legal decision-making emerges as a continuous and interwoven process of emotion and reason (Bergman Blix and Törnqvist).

Another layer of complexity concerns the often unconscious ways emotions shape judicial decisions, particularly regarding the evaluation of defendants' and victims' emotions (van Oorschot, 2023). Johansen, in a Danish context, explores how legal professionals may misinterpret the emotional expressions and communication styles of ethnic minority victims due to unexamined cultural norms that shape courtroom expectations. Expectations for victims to be “calm and quiet” reflect broader Danish cultural schemas that are not neutral but shaped by intersecting factors like race, gender, and class.

## 5 How preconceptions and stereotypical interpretations create and uphold differences within and across legal settings

Although objectivity is often treated as a universal legal ideal, the ways in which emotions and biases are managed in court are deeply shaped by cultural, institutional, and legal contexts. What counts as appropriate emotion, credible testimony, or rational judgment varies across systems. What is considered to be bias is not simply personal but socially constructed and embedded in courtroom practice. This section explores how legal decision-making is influenced by preconceptions about emotional expression, professional conduct, and evidentiary legitimacy and considers how these assumptions help uphold both interpersonal and systemic differences. These dynamics play out differently across national legal cultures—objectivity is always locally made—as the articles here demonstrate.

The concept of judicial neutrality relies on the idea that legal actors can transcend their social and emotional positioning to advance fairness and impartiality. Yet socio-legal research has long argued that emotion and bias are not merely external threats to reason but part of how law functions (Bandes and Blumenthal, 2012; Roach Anleu and Mack, 2017; Grossi, 2015). Rather than being excluded from legal processes, affect and preconceptions shape how cases are interpreted, how parties are evaluated, and how decisions are justified. These effects are not the same everywhere; legal professionals are trained to adopt different emotional styles depending on the culture and structure of their institutions (Bergman Blix and Törnqvist, 2025)

Preconceptions and stereotypical interpretations often operate through tacit norms about emotional appropriateness, which differ depending on the courtroom participant and the surrounding legal culture. As several articles in the Research Topic show, victims' emotional expressions are evaluated against normative standards that are both gendered and culturally specific. For example, in the Swedish context, legal actors are expected to assess credibility with detachment, yet these assessments remain deeply influenced by how well a victim's emotional display fits the expected script of sincere distress (Bladini). Similarly, in the Netherlands, judges manage the inclusion of victim impact statements by controlling the extent to which emotional narratives can be acknowledged without appearing to compromise neutrality (Bosma). In both cases, institutional expectations about proper courtroom emotion produce differential outcomes that reflect broader social hierarchies.

Similarly, preconceptions and stereotypical interpretations also appear in the emotional responses of legal professionals themselves. While judicial neutrality implies emotional self-restraint, maintaining this posture requires substantial affective labor. Judges develop informal strategies, such as heuristics or internal “rules of thumb”, to make difficult or ambiguous decisions feel coherent. In Slovenia, for instance, judges facing inconsistencies in sentencing rely on internal codes that help them justify outcomes, even when formal guidelines fall short (Plesničar). These strategies can provide emotional stability, but they also risk reinforcing normative assumptions about what kinds of defendants, victims, or stories appear consistent, reliable, or deserving.

Importantly, these emotional dynamics are not the same. What appears as proper judicial detachment in one jurisdiction may be interpreted as coldness or inattention in another. In Argentina, lay jurors must learn how to perform objectivity in ways that conform to institutional expectations, even while grappling with their decisions' moral and emotional weight (Amietta). Unlike professional judges, who are trained to regulate emotion as part of their role, jurors must quickly learn to align their affect with courtroom norms. This disjuncture between legal rationality and lay intuition reveals how bias is embedded in individuals and in the emotional expectations that structure institutional roles.

Beyond human actors, technologies used in legal decision-making can encode and reproduce bias. As Contini et al. argue, predictive algorithms and AI-based tools are often framed as impartial, but they draw on data that reflect past inequities and emotional hierarchies. By embedding assumptions about credibility, risk, and emotional appropriateness into their design, these tools risk replicating—even amplifying—the very patterns they are meant to overcome. The aspiration to depersonalize decision-making through automation may, paradoxically, obscure how affective and social values continue to shape legal outcomes.

The increasing integration of generative AI and predictive systems into legal decision-making therefore raises important questions about how objectivity and emotion are conceptualized and operationalized. As this Research Topic suggests, emotion is not peripheral to legal reasoning but central to how objectivity is performed and sustained. Given that AI systems lack the capacity for emotional reflexivity, empathy, or contextual understanding, their use in legal contexts demands critical scrutiny. Future research would benefit from interdisciplinary engagement to explore whether and how technological tools might be developed to acknowledge the epistemic role of emotion without reinforcing patterns of exclusion or oversimplification.

Across these examples, the contributions in the Research Topic demonstrate that preconceptions and stereotypical interpretations are not simply a deviation from objectivity; they are part of how objectivity is produced, stabilized, and performed. They operate not only between individuals but across professional cultures, legal traditions, and national contexts. Whether through empathy, doubt, moral discomfort, or calculated restraint, legal actors engage in emotional work that is shaped by—and helps to reproduce—existing hierarchies. Understanding these dynamics requires us to see objectivity not as a universal standard but as a locally and emotionally negotiated ideal.

## 6 Conclusion

These research articles collectively challenge the traditional dichotomy between law and emotion, illustrating that objectivity in legal contexts is not a detached, universal standard but a socially constructed and emotionally negotiated practice. This perspective aligns with the growing body of scholarship that recognizes emotions as integral to legal processes, shaping and being shaped by institutional norms and cultural contexts.

By examining how emotions influence legal actors' perceptions, decisions, and interactions across various jurisdictions, our contributors highlight the variability and complexity inherent in legal systems. This approach underscores the necessity of understanding law not as a purely rational system but as one deeply embedded in emotional and social frameworks, and reinforces the importance of empirical research.

We are pleased to contribute to this expanding field of inquiry, offering insights that deepen our understanding of the emotional dimensions of law and challenge preconceived notions about legal objectivity and neutrality. As legal systems continue to evolve amidst societal changes, acknowledging and investigating the multidimensional nature of emotion is vital for a comprehensive and equitable understanding of justice and law in action.
